# A Sanitary Taste of the London Wine Docks

**Published:** 1855-12

**Authors:** 


### A SANITARY TASTE OF THE LONDON WINE DOCKS.

A RUMOUR was abroad that, in some of the vaults of the London wine docks, sanitary provisions were exceedingly defective; that ventilation was uncared for; that the air was incessantly surcharged with carbonic acid, watery vapour, and alcoholic spirits; that there was extensive leakage of sewage through the walls of the vaults; and that, as a result of all, the health of the workmen employed in these places was seriously interfered with. These statements, as they were related, bore the full appearance of truth: the particular vaults in which these defective sanitary arrangements most existed were defined; and the diseases, viz. diarrhœa, loss of appetite, emaciation, rheumatism, and pulmonary affections, with which the workmen suffered, were distinctly specified.

Under these circumstances, two volunteer sanitary reporters thought proper to make a visit to these establishments, and confirm or reject the statements thus offered for consideration. Armed with the necessary passport, "a tasting order", they made, therefore, a careful inspection of the vaults against which the main charges had been made. It is right to premise that many of these charges were unfounded.

The reporters remark incidentally that a more interesting sanitary inspection could not well be conceived than the one in question, especially when the observer is armed with the passport referred to. The vaults visited were the East vault, the Crescent vault, and No. 3. Early in their visit, they had the advantage of meeting and making the acquaintance of Inspector Reakon, who exhibited a large smack of scientific learning, explained fluently the principle of endosmosis through wine casks, and said a great many good things, which there is no time to repeat. They met, too, with an intelligent workman, who, after giving a profound opinion on a certain specimen of young sherry, under the influence of which he threw off much of that gloominess incident to these regions of Avernus, invited them pressingly to accompany him to hear an address "On the deleterious effects of spirituous liquors on the human frame"; which address he, in the character of President, was about to deliver from the chair, that self-same evening, to the learned the members of the Lancasterian Institution, Church Street, Stoke Newington. The offer was tempting to the last degree; for the professor was evidently getting up his subject with great care, and was possibly preparing to illustrate it practically. Unhappily, owing to other engagements, the invitation was reluctantly declined.

Returning to the vaults: their ventilation is indifferently provided for. In the eleven acres of the East vault there are no shafts for ventilation; but there are in different parts six or seven holes, perhaps a foot square, opening transversely, and grated. That there is little or no current of air through the vault is clear from the fact that the light fungous growths, peculiar to the roofs of these places, and which are pointed out to visitors for special admiration, are never seen to stir under ordinary circumstances, although the slightest breath blown towards them puts them into immediate vibration. The atmosphere is close, faint, earthy, and in itself half intoxicating to the stranger. The lamps by which the place is lighted burn dismally, and show a frequent unhappy tendency to give up the ghost. The walls are damp: but the history of the sewage leakage did not seem to be correct; at all events, the visitors saw no trace of it. In some places there was an accumulation of filthy moist matter on the roofs; but it was found that this was principally confined to parts where the surface over the vaults was exposed freely to the air, and where, the roof being thus extensively cooled above, the condensation of water from the vault on its interior surface was easily, and, as Inspector Reakon remarked, "philosophically" accounted for. But, in the absence of foul air arising from the evaporation of sewage, there is sufficient impurity of atmosphere to account for, and to produce necessarily, a serious amount of ill-health amongst the workmen. The reporters decidedly think, that where lamps burn dim, and go out without ceremony, men cannot live eight hours a day with impunity; and, from the facts they were enabled to extract, there can be no doubt that the men employed suffer very soon from the effects of their position—that they contract asthmatic coughs, lose appetite, become emaciated, and see the end of life prematurely. They met, however, with one man, who stated that he had worked in the vault regularly for twenty-seven years, and had felt little inconvenience. Whether men stood the work well or ill, depended, according to the long experience of this authority, "more on the constitootion than the wault". The habits of the men, whether temperate or not, have of course much to do with the result of the occupation. Where the temptation is very strong to imbibe good wine by the mouth, the opportunity to do so will not be lost because the lungs are taking in the same agent in the form of vapour. There is thus administered in some cases a double dose of the wine poison.

If it be a fact, and the writers do not dispute it, that one man has worked in these vaults twenty-seven years, they consider it a remarkable exception to the general rule; for, without being dictatorial, they have reason to believe that, on an average, ten years of constant occupation in the wine vault are more than sufficient to impair materially, and often completely, the health of the workman.

In considering the plans that might be adopted for remedying the evil effects arising from this employment, many difficulties have to be encountered. If thousands of hogsheads of wine must be housed, and doctored, and ripened, and bottled, vast numbers of men are necessary for the work; and, for commercial reasons, certain proceedings must be followed, which are not compatible with stringent sanitary regulations. Free ventilation through a wine vault, so as to keep it charged with fresh dry air, would lead to a serious loss of wine by evaporation. A certain fixed temperature, about 60° Fahr., which could not be sustained if free currents of air passed through the storehouse, is an essential in these establishments; and not less so is a certain proportion of aqueous vapour. In plain terms, the wine must be buried with a certain set series of ceremonials; and as wine, like many other things strictly material, is of more value in the money market than healthy human bodies, which are something more than material, and as the laws of the money market are indisputable, the man and the wine are put in the balance together, and the man kicks the beam.

The directors of the London wine docks are possibly "practical men", who have no terrors of wine, and who would treat as it deserves any theoretical hint that should refer to new plans for the stowage, the doctoring, and the ripening of the juice of the grape. The reporters would therefore decline to dwell on this point, even had they a suggestion of importance to offer. But they would ask these gentlemen a really practical question: Would they not, in their own persons, find it rather too hard a fate to be buried for eight mortal hours a day in a damp, dark cellar, where carbonic acid gas, water vapour, and gaseous wine, stand as three to one to common air?

